# Case report: One case of refractory membranous nephropathy with hypokalemia after rituximab infusion was switched to obinutuzumab without recurrence of hypokalemia

**DOI:** 10.3389/fphar.2024.1347880

**Published:** 2024-01-26

**Authors:** Yao Zhang, Jing Sun, Jie Gao, Weiyan Sun, Liang Xu, Chunjuan Zhai, WenYan Su, Haiping Wang

**Affiliations:** ^1^ Department of Nephrology, Shandong Provincial Hospital Affiliated to Shandong First Medical University, Jinan, China; ^2^ The Peoples Hospital of Huaiyin, Jinan, China; ^3^ Department of Cardiology Shandong Provincial Hospital Affiliated to Shandong First Medical University, Jinan, China

**Keywords:** adverse drug reactions, rituximab, hypokalemia, PLA2R-Ab-associated membranous nephropathy, obinutuzumab, case report

## Abstract

Rituximab (RTX) is a monoclonal antibody commonly used to treat PLA2R-associated membranous nephropathy (MN). This report presents a case of refractory MN in a patient who experienced severe hypokalemia, a rare but clinically significant condition, after the 5th RTX infusion. Clinicians should be aware of the potential for hypokalemia and its management during or after RTX infusion. After the onset of hypokalemia, the patient received treatment with obinutuzumab and achieved partial remission of renal disease without experiencing further hypokalemia. Obinutuzumab may be a viable alternative therapy for refractory membranous nephropathy that develops side effects after rituximab therapy or is refractory to it, but further studies are necessary to determine its efficacy and safety.

## Introduction

Idiopathic membranous nephropathy (IMN) is the most common cause of primary nephrotic syndrome among adults, accounting for approximately 20%–30% of cases (Ronco et al., 2021). It primarily affects individuals aged 30 to 50, with a slightly higher incidence in males. Up to 80% of MN patients have autoantibodies to M-type phospholipase A2 receptor (PLA2R) in their bloodstream. Additional autoantibodies, such as thrombospondin type-1 domain-containing 7A (THSD7A) (Couser, 2017), are increasingly being recognized. The progression of IMN varies among individuals. Some patients may experience a self-limiting disease course, with spontaneous remission occurring in approximately one-third of cases. However, others may develop persistent nephrotic syndrome. In cases of persistent nephrotic syndrome (NS), up to 40% of patients may progress to kidney failure over the course of 10 years (Caravaca-Fontan et al., 2022). For patients with this condition, immunosuppressive therapy is recommended to improve long-term kidney outcomes. Treatment options include corticosteroids, alkylating agents like cyclophosphamide, calcineurin inhibitors such as cyclosporine and tacrolimus, and rituximab, an anti-CD20 monoclonal antibody.

The use of anti-CD20 antibodies has shown promise in treating idiopathic membranous nephropathy (IMN). Rituximab (RTX), a chimeric antibody that targets CD20, a surface antigen found on B-cells that regulates their growth and differentiation, has demonstrated potential in this regard. With increased and prolonged usage, more information has been gathered regarding its potential adverse effects. Adverse reaction reports indicate that most cases of adverse reactions occur within 20–40 min following intravenous infusion. However, some adverse reactions may occur between 90 min and 1 month after drug administration, even after the injection is completed. It is important to note that these side effects can occur at any time after the injection. Possible side effects of RTX include infusion-related reactions, infections, progressive multifocal leukoencephalopathy (PML), hypersensitivity reactions, and heart problems (Paul and Cartron, 2019; Basu et al., 2022).

We present a case of hypokalaemia in a patient with refractory membranous nephropathy following the fifth RTX infusion. The patient was subsequently treated with Obinutuzumab, a humanized type II anti-CD20 monoclonal antibody that targets a different epitope on CD20 than rituximab.

### Case description

**FIGURE 1:**
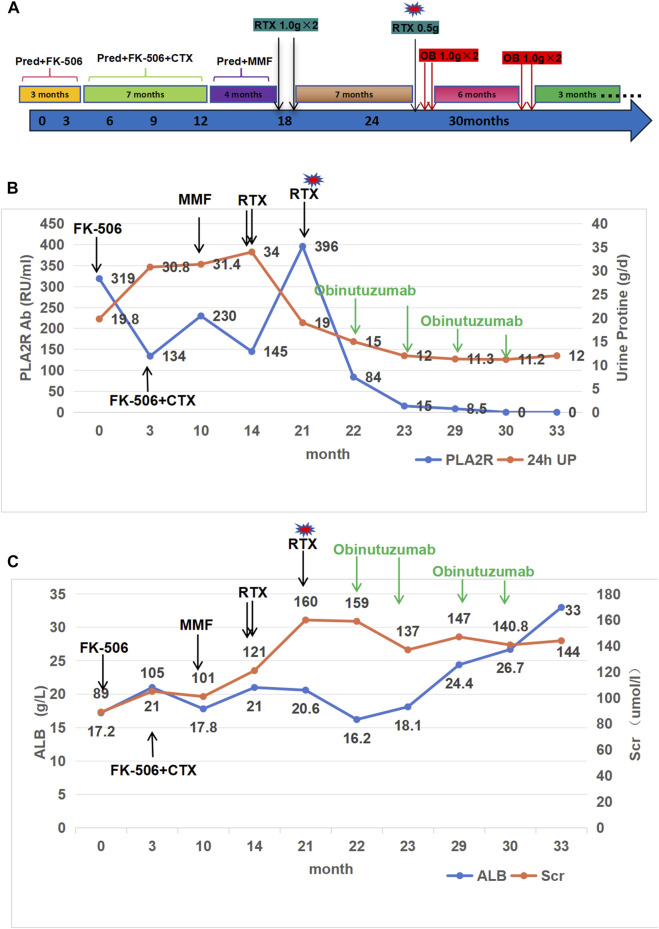
Clinical course, proteinuria, phospholipase A2 receptor antibody (PLA2R Ab), albumin and creatinine level in the case. **(A)**: Historical view of immunosuppressive treatment with a detail on rituximab and obinutuzumab administrations. Pred, prednisone; CTX, cyclophosphamide; Fk-506, tacrolimus; MMF, mycophenolate mofetil; RTX, rituximab; OB, obinutuzumab. **(B)**: Proteinuria and PLA2R Ab trends in relation to the treatments. **(C)**: creatinine (Scr) and albumin (Alb) trends in relation to the treatments.

In December 2020, a 31-year-old man presented with NS and was subsequently diagnosed with PLA2R-associated MN after a kidney biopsy. Secondary screenings for viruses (HBV, HCV and HIV) and autoimmune diseases (specifically ANA) were negative. At the time of biopsy, his serum creatinine (Scr) was 89.1 μmol/L, urine protein (UP)was 19.8 g/d, and serum albumin was 17.2 g/L. Furthermore, the PLA2R antibody titer was 319 RU/mL. The biopsy also yielded a positive PLA2R stain. The patient was initially treated with tacrolimus 1 mg twice daily in combination with prednisone 30 mg once daily. After 2 weeks, the tacrolimus dose was increased to 2 mg twice daily due to low blood concentration. Despite 3 months of treatment, the patient remained unresponsive. An additional 7 months of therapy comprising prednisone, tacrolimus, and oral cyclophosphamide (total dose of 12 g) was administered, but proved to be ineffective. After completing the treatment, the patient’s serum albumin level was 18.2 g/L, urinary protein level was 24.4 g/d, Scr level was 121 umol/L, and PLA2R antibody titre was 230 RU/mL. Despite 4 months of subsequent MMF therapy, the patient did not respond and was given two intravenous doses of RTX (1 g × 2), 2 weeks apart (Figure 1A). Before each rituximab infusion, the patient was given 300 mg of oral acetaminophen, 25 mg of intramuscular promethazine, and 40 mg of intravenous methylprednisolone, as instructed by the manufacturer, to prevent infusion reactions. The patient did not report any discomfort during or after the RTX infusion.

**FIGURE 2:**
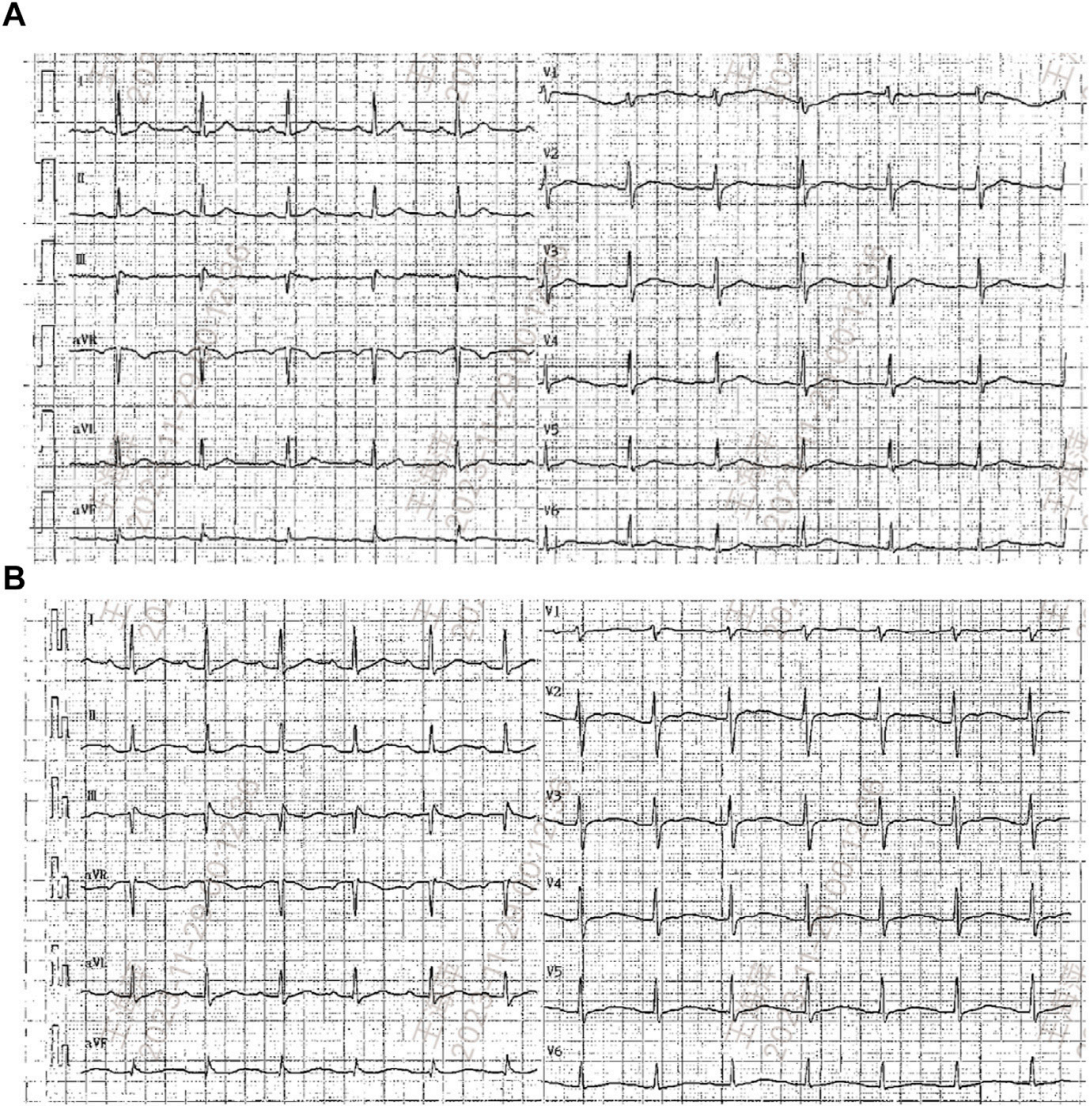
Electrocardiograms (ECG) before **(A)** and after rituximab administration when hypokalemia occurred **(B)**.

Seven months later, the patient’s Scr level was recorded at 160umol/L, serum albumin at 20.6 g/L, and UP at 19 g/d, with a PLA2R antibody titer of 396 RU/mL (Figures 1B, C). On 20 August 2022, the patient is scheduled to receive a fifth intravenous dose of rituximab (375 mg/m2, total dose of 500 mg) while admitted as an inpatient in our Nephrology unit. Before starting the infusion at 7a.m., a blood test was conducted to check the patient’s serum potassium level, which was found to be 3.59 mmol/L. The levels of sodium, magnesium, glucose, calcium and carbon dioxide binding capacity (CO2-CP) were normal. After 5 h of infusion, the patient reported weakness in the left little finger and ring finger when lifting. The infusion was paused for 1 h and resumed only after the patient reported no significant change. The infusion was completed 1.5 h later. Thirty minutes after the infusion, the patient experienced weakness in the lower limbs and left upper limb, along with post-activity palpitations. The electrocardiogram (ECG) displayed a sinus rhythm with a heart rate of 79 bpm. Additionally, it showed prolonged QT (U) and PR intervals, flattened T waves, and chest leads indicating U waves and T-U fusion waves (Figure 2). The patient’s blood glucose level was measured and found to be 9.2 mmol/L. Blood electrolytes were analyzed urgently. The patient experienced weakness in both hips and had difficulty getting out of bed. With the assistance of a family member, the patient gradually sat down on the floor due to weakened lower limbs. The patient did not report any headache or dizziness. The blood pressure was 161/88 mmHg, the heart rate was 75 beats per minute, and the oxygen saturation (SPO2) was 97%. Emergency blood tests showed a potassium level of 1.86 mmol/L, blood glucose of 6.87 mmol/L, blood calcium of 2.13 mmol/L, blood sodium of 138.3 mmol/L, and CO2-CP of 23.2 mmol/L. The patient was administered 500 mL of saline and 15 mL of 10% potassium chloride (KCl) intravenously, and was also given potassium citrate granules orally. After 7 h, the patient reported a decrease in lower limb weakness. After 18 h, a potassium recheck showed a level of 2.43 mmol/L. An additional 15 mL of 10% KCl was administered intravenously, while oral potassium citrate granules were continued to supplement potassium. At the 22-h mark, the patient’s potassium level was rechecked and found to be 3.94 mmol/L, allowing him to resume normal activities.

**FIGURE 3:**
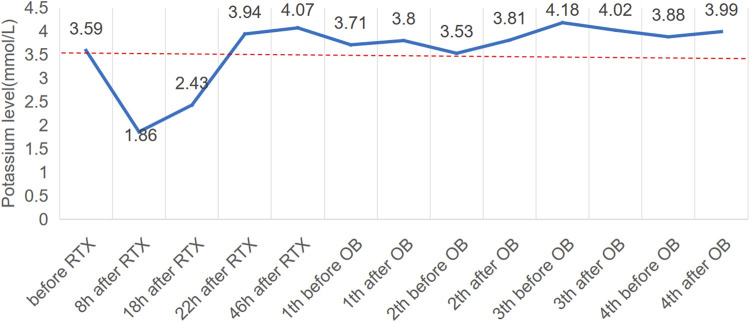
Potassium levels (mmol/L) over time and potassium central laboratory normal value (3.5 mmol/L, dotted line). RTX, rituximab; OB, obinutuzumab.

The patient’s treatment was changed to obinutuzumab after 3 weeks due to an adverse reaction of hypokalemia following the RTX infusion and the patient’s ongoing renal disease. The patient received 2.0 g of obinutuzumab over the course of 1 month, and no infusion-related adverse reactions, including hypokalemia, were observed. In May 2023 (Figure 3), the patient’s proteinuria decreased by 50%, with a quantitative 24-h urine protein level of 11 g and a blood albumin level of 32.5 g/L (the highest recorded since treatment). The patient’s PLA2R Ab titer was also significantly reduced to 15 RU/mL, and renal function remained stable with a Scr level of 147umol/l. Additionally, the patient received another 2.0 g of obinutuzumab in May and June 2023. As of September 2023, the patient achieved partial remission with a 24-h urine protein level of 9 g, a serum albumin level of 34.1 g/L, and a serum creatinine level of 136 umol/L.

## Discussion

We present a case of PLA2R-Ab-associated membranous nephropathy (MN) in a patient who was unresponsive to multiple treatments, including prednisolone, tacrolimus, cyclophosphamide, and mycophenolate mofetil. The patient achieved partial remission of his nephropathy after receiving successive applications of CD20 monoclonal antibodies, specifically rituximab and obituzumab. During the fifth infusion of rituximab, the patient experienced infusion-related hypokalemia, but did not experience any infusion reactions or hypokalemia after switching to obinutuzumab.

In this patient, the PLA2R antibody titers increased from 145 to 396 after treatment with 2.0 g of rituximab. Although a decreasing trend in PLA2R and urinary proteins was observed with the subsequent addition of 0.5 g of RTX, the patient developed hypokalemia while receiving the last 0.5 g of RTX. Therefore, we had to interrupt the rituximab treatment, which also affected the judgment of whether RTX was ultimately effective in this patient. Regarding the consideration of the cause of hypokalemia, the patient was not taking any diuretics at that time. Prior to each infusion of rituximab, we followed the manufacturer’s instructions and administered 300 mg of oral acetaminophen, 25 mg of intramuscular ipecac, and 40 mg of intravenous methylprednisolone to prevent infusion reactions. Glucocorticoids are the drugs most likely to cause hypokalemia prior to rituximab infusion (Kokot and Hyla-Klekot, 2008). Glucocorticoids can cause hypokalemia through multiple mechanisms. Firstly, they increase the activity of the Na + -K+ enzyme in skeletal muscle cells, resulting in an influx of potassium ions into the cells. Additionally, they have a mild mineralocorticoid effect, leading to sodium retention and potassium excretion (Ben et al., 2009). Prolonged use of glucocorticoids can result in excessive potassium loss. However, hypokalemia resulting from the potassium-excreting effects of glucocorticoids is a chronic and cumulative process. The incidence of hypokalemia is proportional to the range, duration, and dose of glucocorticoid action. In general, routine doses do not result in a sudden drop in serum potassium. Prior to the infusion of RTX, a routine dose of methylprednisolone 40 mg was administered intravenously to patients with nephrotic syndrome. Although glucocorticoid-induced hypokalemia cannot be completely ruled out, it is not considered a significant contributing factor.

However, treatment with rituximab may cause electrolyte imbalances, such as hypokalemia. The literature to date only reports three cases of hypokalemia resulting from rituximab administration. A single young patient with recurring minimal change disease (MCD) experienced hypokalaemia after her sixth rituximab dose (Guzzi et al., 2020). An elderly patient with MCD, who received rituximab *in lieu* of steroid therapy, developed hypokalaemia after only her third dose. Additionally, a young woman with idiopathic nephrotic syndrome (NS) was introduced to rituximab as a means of decreasing reliance on prednisone (Song et al., 2023). The diagnosis of acute hypokalaemia was made during her sixth intravenous rituximab infusion. The three patients presented with symptoms of dizziness, palpitations, fatigue and muscle spasms in the lower limbs. The medical professionals quickly identified the symptoms, and hypokalemia was diagnosed through blood tests. Intravenous and oral potassium supplementation were administered, and hypokalemia was successfully resolved without any negative after-effects. Our patient initially exhibited fatigue and weakness, which worsened after receiving the infusion. An urgent blood test confirmed the diagnosis of hypokalaemia. After receiving potassium supplements, the patient’s blood potassium levels returned to normal and their symptoms abated.

Although three occurrences of rituximab-induced hypokalemia have been reported to date, the precise mechanism behind hypokalemia triggered by rituximab remains unknown. Among rituximab-related adverse events, infusion-related adverse events were the most common, with the most frequently observed clinical manifestations including fever, chills, asthenia, headache, anaphylaxis, anaphylactoid events, bronchospasm, dyspnea, rash, urticaria, angioedema, or pruritus. These clinical manifestations are usually associated with the first infusion and the onset is usually around 30–120 min (Wong and Long, 2017). These adverse reactions are considered to be hypersensitivity reactions, and treatment with desensitizing medications has been shown to be effective (Sloane et al., 2016; Isabwe et al., 2018). However, based on our and two other reports in the literature, none of these hypokalemia associated with rituximab infusion occurred at the time of the first infusion, and therefore, speculation about the mechanism of immune-mediated hypersensitivity reactions behind this adverse reaction seems unlikely. Indeed, the mechanism by which rituximab infusion induces hypokalemia is currently unknown. Additional experimental data should be provided to elucidate the relationship between rituximab and hypokalemia. A study of patients with papular urticaria treated with rituximab found that the amplification of KCNN4 potassium channels on the surface of B cells occurred. However, it is unlikely that hypokalemia is due to their activation since B-cell KCNN4 channels inadvertently increase calcium ion influx by releasing potassium (Caillot et al., 2018). Studies have confirmed that rituximab effectively reduces intracellular Ca2+ levels and blocks intermediate conductance Ca2+-activated K(IK) channels (Wang et al., 2007). In addition to complement-dependent cytotoxicity, rituximab also stimulates FcγRIIB receptors and inhibits Kv1.3 channels, which leads to apoptosis in malignant B lymphocytes (Wang et al., 2012). Analyzing the effects of antineoplastic drugs on electrolyte imbalance could inform future exploration of the relationship between rituximab and hypokalemia. For instance, platinum compounds have been linked to imbalances in sodium, potassium, and magnesium, while alkylating agents and periwinkle alkaloids may cause hyponatremia due to the syndrome of inappropriate antidiuretic hormone secretion (SIADH) (Verzicco et al., 2020). Targeted therapies, including monoclonal antibodies, tyrosine kinase inhibitors, immunomodulators, and mammalian target of rapamycin, have the potential to cause hyponatremia associated with SIADH and, to a lesser extent, urinary sodium loss (Jhaveri et al., 2017). Anti-epidermal growth factor receptor (EGFR) antibodies may cause significant magnesium and potassium losses in clinical settings. SAKURADA recommends monitoring serum electrolytes for up to 8 weeks after anti-EGFR antibody dosing (2016).

None of the three patients mentioned in the literature reported whether rituximab was continued following hypokalemia development. It is unclear whether continued use of rituximab will result in the reoccurrence of hypokalemia. To avoid a recurrence of this dangerous condition, we decided not to continue rituximab after consulting with the pharmacist. The decision to switch to obinutuzumab was based on the fact that, although both rituximab and obinutuzumab bind to the CD20 antigen on B cells, their mechanisms of action and side effects differ slightly due to structural differences and interactions with the immune system. Obinutuzumab, a humanized type II anti-CD20 monoclonal antibody (Basu et al., 2022), has been found to be effective in treating refractory PLA2R-related MN that does not respond to prednisolone, cyclosporine, cyclophosphamide, or rituximab (Sethi et al., 2020; Naik et al., 2023; Conversano et al., 2024). Compared to rituximab, obinutuzumab is a more potent inducer of antibody-dependent cell-mediated cytotoxicity (ADCC) and direct cell death (DCD). Obinutuzumab is believed to lead to a deeper and more enduring depletion of the body’s B-cell population and has shown effectiveness in cases where rituximab has failed (Klomjit et al., 2020; Hudson et al., 2022). However, there is ongoing debate regarding the adverse effects or toxicity of obinutuzumab compared to rituximab. Some studies suggest a high rate of toxicity associated with the use of obinutuzumab. However, Naomi A (Amudala et al., 2022) documented three cases of ANCA-associated vasculitis patients who were treated with obinutuzumab after being allergic to rituximab. Despite prior anaphylactic reactions to rituximab, all patients tolerated obinutuzumab well and maintained remission. In our case, we switched to obinutuzumab after correcting the hypokalemia, which resulted in partial remission and the absence of hypokalemia recurrence. However, it is crucial to avoid making broad conclusions based on a limited case series. Further research is needed to determine the safety profile of obinutuzumab.

The clinical features of hypokalemia can range from mild to severe, depending on the degree of potassium decline. Common symptoms associated with this condition include muscle weakness, fatigue, palpitations, muscle cramps, and cardiac arrhythmias. Severe hypokalemia can result in life-threatening complications such as paralysis and cardiac arrest. It is important to consider hypokalemia as a potential complication during RTX therapy. Although RTX has shown effectiveness in treating various medical conditions (Deng and Xu, 2023), it is important to consider the risk of hypokalemia. Vigilant monitoring of potassium levels, prompt intervention when necessary, and appropriate potassium replacement are vital to ensure the safe and successful management of patients receiving RTX therapy. In cases where hypokalemia is corrected, continued treatment with obinutuzumab can also be attempted.

In conclusion, it is essential for clinicians to be aware of the potential risk of hypokalemia during RTX therapy and its management. This knowledge will enable them to implement preventive measures and initiate appropriate treatment.

## Data Availability

The original contributions presented in the study are included in the article/Supplementary material, further inquiries can be directed to the corresponding author.
